# Hybrid BOTDA-DIC sensing for flexural behavior characterization of eco-concrete reinforced beams under four-point loading

**DOI:** 10.1038/s41598-026-53187-y

**Published:** 2026-05-22

**Authors:** Nuraziz Handika, Daral Suraedi, Jessica Sjah, Abdul Halim Hamdany, Bastian Okto Bangkit Sentosa

**Affiliations:** Department of Civil and Environmental Engineering, Faculty of Engineering, Universitas Indonesia, Depok, 16424 Indonesia

**Keywords:** BOTDA, Digital image correlation, Reinforced concrete, Distributed strain, Deflection, POBA, Nano-silica, Engineering, Materials science

## Abstract

This study investigates the performance of Brillouin Optical Time Domain Analysis (BOTDA) and Digital Image Correlation (DIC) for monitoring the flexural behavior of two reinforced concrete (RC) beams constructed using eco-concrete containing Palm Oil Boiler Ash (POBA) and nano-silica as partial cement replacement. Two simply supported beams (300 cm × 15 cm × 25 cm) with different concrete compressive strengths were tested under four-point bending until failure to evaluate the sensing performance under varying structural responses. Distributed strain from BOTDA and full-field displacement from DIC were compared with mid-span deflection measured by Linear Variable Differential Transformers (LVDTs). Both sensing systems successfully captured strain development and stiffness degradation. BOTDA recorded the progressive increase of tensile strain at the bottom fiber, reaching 10,000–18,000 µε, as cracking advanced, while DIC identified multiple flexural cracks with openings ranging from 0.05 to 3.28 mm. BOTDA-derived deflection showed good agreement with LVDT and DIC, with differences less than 7%. The findings highlight the complementary advantages of both systems: BOTDA provides distributed and continuous strain monitoring for global behavior, while DIC captures localized deformation and crack development with high precision. Together, these techniques offer a complementary and reliable approach for detailed structural assessment of reinforced concrete beams.

## Introduction

 Monitoring and assessing the behavior of reinforced concrete (RC) beams under flexural loading is essential for understanding load deflection characteristics, damage mechanism, cracking mechanisms, stiffness degradation, and ultimate failure modes. Experimental studies provide valuable benchmark data that support analytical model calibration, design code development, and validation of new construction materials. Conventional instrumentation, such as strain gauges and Linear Variable Differential Transformers (LVDT), remains widely adopted due to their accuracy and stability. However, these systems offer measurements only at discrete points, which limits their ability to capture distributed strain profiles and localized deformation variations along the investigated structural member^1,2^.

Advancements in structural health monitoring have led to increasing use of optical sensing methods to address these limitations. Brillouin Optical Time Domain Analysis (BOTDA) enables distributed strain sensing along optical fibers over long distances, providing continuous strain profiles rather than isolated measurements. This makes BOTDA highly attractive for observing global deformation behavior, identifying the onset of cracking, and locating strain concentrations associated with stiffness degradation^3^. However, BOTDA exhibits, typically, spatial resolution between 5 and 10 cm, which may limit its ability to detect sudden localized gradients near cracks or reinforcement interfaces^4–6^. Even so, recent applications using fiber sensing textiles in bridge girders demonstrate the potential of BOTDA to capture strain development patterns that would otherwise be missed using traditional sensors^7^.

In parallel, Digital Image Correlation (DIC) has also become an established technique for laboratory-scale experimental mechanics, offering full-field, non-contact measurements of displacement and strain with sub-pixel measurement accuracy. DIC is particularly effective for visualizing crack initiation, propagation, spacing, and strain concentration during flexure in simple prismatic beams^8,9^. Further application also for reinforced concrete elements including beams, slabs, and strengthened members^10–15^. While DIC offers high-resolution surface response measurements, its performance depends on line-of-sight visibility and consistent lighting. Moreover, DIC cannot directly capture internal reinforcement strain, which is required to observe tension stiffening behavior after cracking. BOTDA, in contrast, can be attached to reinforcement or embedded within the concrete surface. However, when used to estimate displacement, BOTDA requires curvature-based strain reconstruction, which may introduce integration uncertainty.

Recent reviews have suggested that hybrid or comparative use of BOTDA and DIC combines the strengths of distributed global sensing and localized high-resolution measurement, offering a more comprehensive method for experimental mechanics^16–18^. Despite these advancements, direct experimental comparisons of BOTDA and DIC applied simultaneously to RC beam failure tests are still limited. Establishing such comparisons is necessary to evaluate measurement correlation, system sensitivity, and suitability for full-scale structural applications.

In parallel with sensing technology advancements, there has been increasing focus on reducing the environmental impact of concrete production. Ordinary Portland Cement contributes significantly to global carbon emissions, driving researchers to explore alternative supplementary cementitious materials. The concrete mix employed in this study, designated CP19S1, incorporates 19% Palm Oil Boiler Ash (POBA) and 1% nano silica as partial cement replacement^19^. POBA is an agricultural by-product with pozzolanic reactivity potential, while nano silica improves packing density and microstructure refinement. Previous studies have confirmed that this mix achieves adequate compressive strength and mechanical performance, indicating its feasibility as an eco-concrete option. However, limited research has examined its structural-scale behavior or its interaction with advanced optical monitoring systems during flexural loading.

This study presents an experimental investigation comparing BOTDA and DIC measurements on two RC beams cast with CP19S1 concrete and tested under four-point bending until failure. The two beams exhibited different compressive strengths, resulting in distinct structural responses, and were used to assess the sensing performance under varying material conditions rather than as directly comparable specimens. Strain profiles obtained from BOTDA method were converted into displacement through curvature integration following^20^ and compared against midspan LVDT readings and DIC measurements. The primary novelty of this work lies in the direct comparison of BOTDA and DIC sensing performance throughout the loading process up to structural failure, while simultaneously evaluating the flexural behavior of a sustainable concrete system incorporating agricultural and nano material components. The combined interpretation of distributed and full-field measurements offers new insight into the complementary strengths of both sensing methods. The findings contribute to expanding the use of optical sensing in laboratory and field monitoring of RC structures, while also advancing understanding of the behavior of eco-concrete materials monitored using advanced optical sensing techniques.

## Experimental section

### Specimen, materials, and mix design

**Fig. 1:**
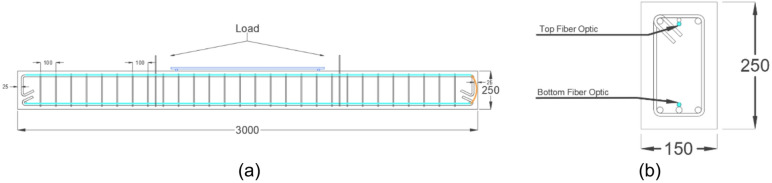
Geometry and BOTDA optical fiber layout of the RC beam specimen: (**a**) side view; (**b**) cross-sectional view showing optical fibers installed along the longitudinal reinforcement bars and mechanically fixed using cable ties prior to concrete casting.

**Fig. 2:**
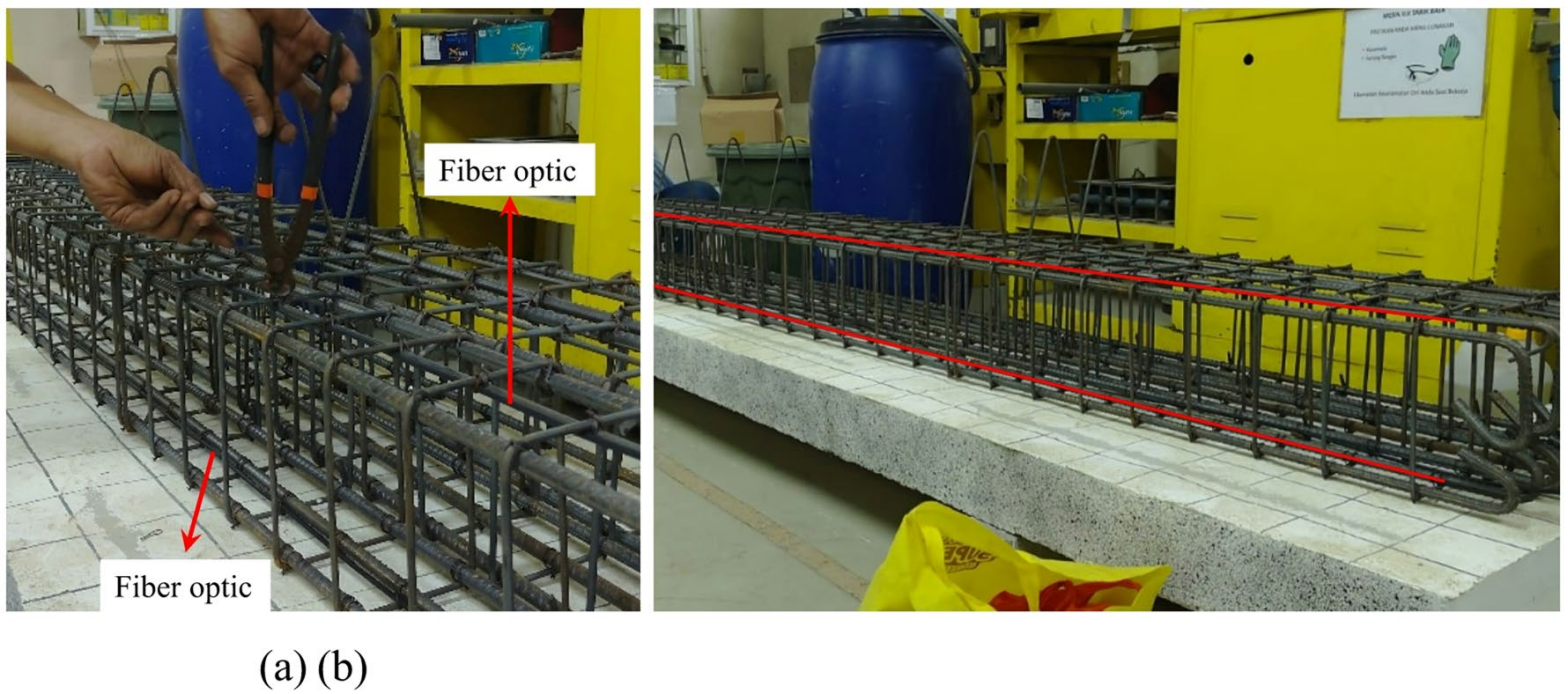
Installation of the BOTDA optical fiber installation on the reinforcement cage: (**a**) BOTDA optical fiber is black cable indicated by red arrows, (**b**) Reinforcement cage of the RC beam (The fiber optic cable was attached along the reinforcing bars, as indicated by the red line).

The test specimens were reinforced concrete (RC) beams with dimensions of 3000 mm in length, 150 mm in width, and 250 mm in depth. The reinforcement layout was designed to promote flexural rather than shear failure, as shown in Figs. 1 and 2. The beams were reinforced with two D13 deformed steel bars at the tension face and two D13 deformed steel bars at the compression face. Shear reinforcement was provided using Ø6 mm plain round steel stirrups spaced at 150 mm center-to-center. The longitudinal reinforcement consisted of deformed steel bars with a nominal yield strength of fy = 420 MPa, while the transverse reinforcement (stirrups) had a yield strength of fyt = 280 MPa. Only the fibers attached to the reinforcement bars were used for BOTDA strain measurement and analysis in this study.

**Fig. 3:**
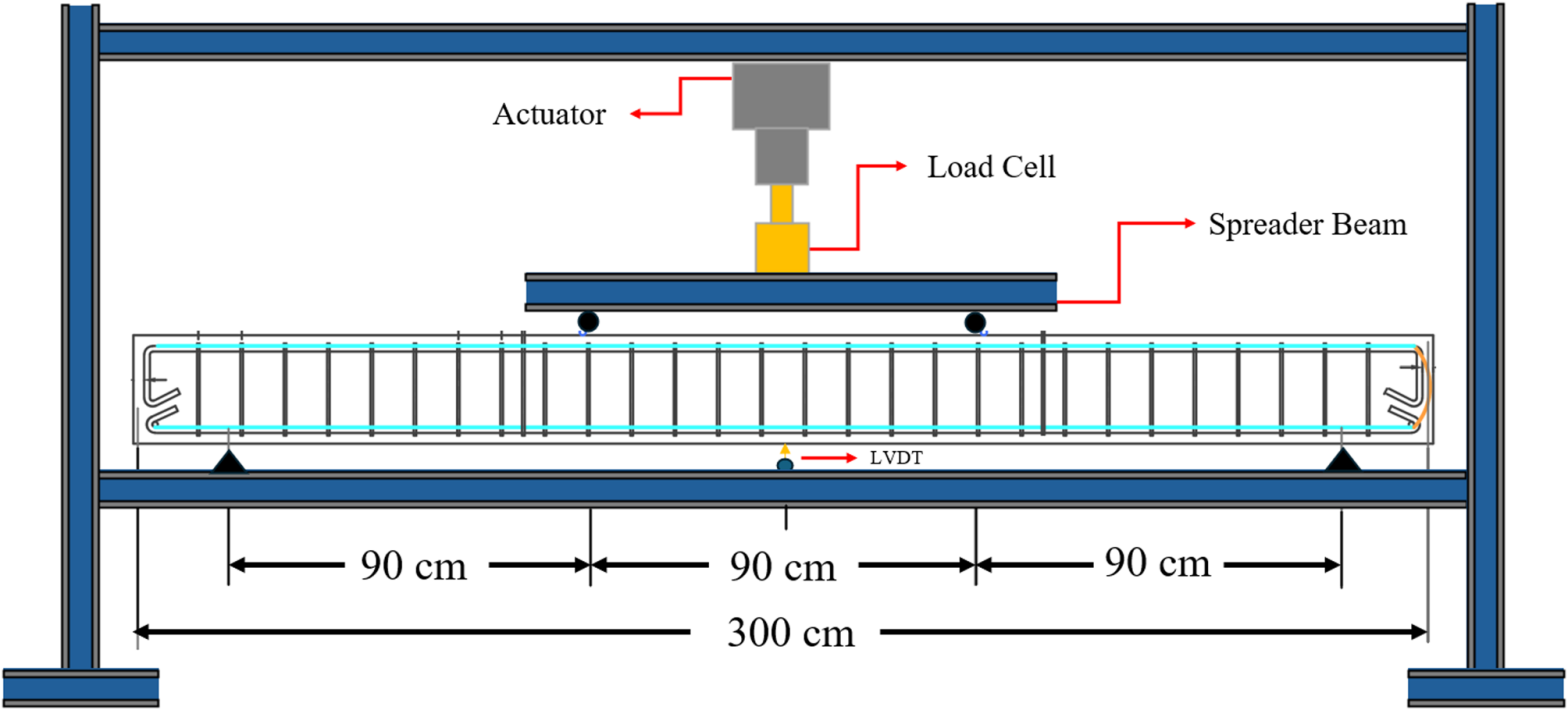
Schematic of the four-point bending test setup showing the actuator, load cell, spreader beam, pin-roller supports, mid-span LVDT, and beam geometry.

Figure 3 illustrates the schematic configuration of the four-point bending test. The reinforced concrete beam, with a total length of 300 cm, was simply supported using a pin support at one end and a roller support at the other. Two concentrated loads were applied symmetrically through a steel spreader beam connected to a hydraulic actuator and monitored using a load cell, creating a constant-moment region at mid-span. The distance between the supports and loading points were each 90 cm. A Linear Variable Differential Transformer (LVDT) was installed at the mid-span soffit of the beam to measure vertical deflection during loading.

**Fig. 4:**
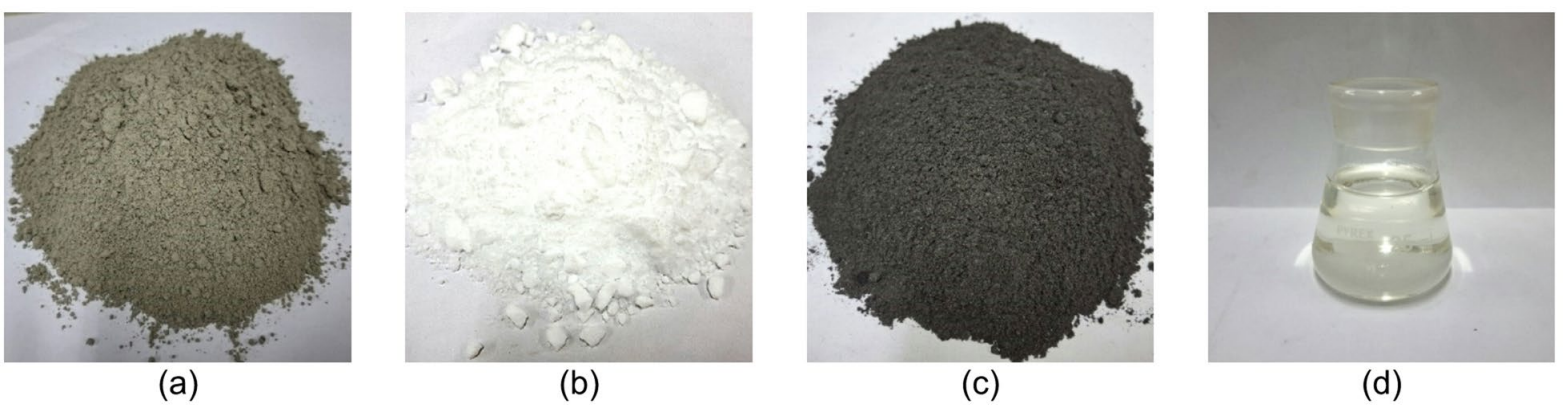
(**a**) Ordinary Portland Cement (OPC), (**b**) Nano silica (nSiO_2_), (**c**) Palm oil boiler ash (POBA), (**d**) Polycarboxylate superplasticizer (PCE).

The CP19S1 mix was adopted based on previous studies showing that a 19% replacement of cement with POBA offers a balance between sustainability and mechanical performance, while the addition of 1% nano-silica improves microstructural densification and strength development^19,21^. In this mixture, 19% POBA and 1% nano-silica (nSiO₂) were incorporated as a partial cement replacement and as an additive, respectively. A polycarboxylate-ether (PCE) based superplasticizer was included to enhance workability and maintain a constant water–cement ratio. The mix design followed the ACI 211.1–22 guidelines, and the detailed mix composition is presented in Table 1 (Fig. 4).

**Table 1:** Mix proportion of CP19S1 eco-concrete.

Material	Quantity
OPC (ordinary portland cement)	371.06 kg
POBA	88.13 kg
Nano-silica (nSiO_2_)	4.64 kg
Superplasticizer (PCE)	2.32 L
Water	177 L
Coarse aggregate	1163.9 kg
Fine aggregate (sand)	709.7 kg
Water–cement ratio (w/c)	0.48

The fresh concrete was mechanically mixed, poured into steel molds, and compacted using a vibrating table to minimize air voids. Standard curing in water was performed prior to testing. Companion cylinder specimens (150 mm × 300 mm) were cast and tested in compression to determine the concrete strength. Although both beams were produced using the same CP19S1 mix design, they were cast under different environmental conditions during mixing and early curing. Variations in ambient temperature and humidity during this stage influenced strength development, resulting in different compressive strengths between Beam 1 and Beam 2. Therefore, the two specimens represent distinct material conditions rather than directly comparable replicates. The results showed average compressive strengths of 43.27 MPa (Beam 1) and 25.41 MPa (Beam 2) at 31 days, reflecting variability in curing conditions and strength development.

### Test setup and loading

The reinforced concrete (RC) beams were tested under a four-point bending configuration with simply supported ends, following the procedure outlined in ASTM D6272-17. Each beam specimen measured 3000 mm in total length, 150 mm in width, and 250 mm in depth. Two concentrated loads were applied symmetrically at one-third spans, creating a pure moment bending area in the central portion of the beam. This configuration was selected to promote flexural failure and ensure a uniform bending moment in the mid-span zone, where the BOTDA and DIC measurements were concentrated.

Each beam was supported by roller and pin supports to simulate simply supported boundary conditions. The load was applied under quasi-static conditions using a hydraulic jack and transferred through a steel spreader beam to ensure equal force distribution at the two loading points. The loading followed a predefined cyclic protocol, applied in controlled incremental stages from 0 up to a peak load of 1200 kgf. This value excludes the self-weight of the spreader beam (~ 30 kgf).

Beam 2 was tested prior to Beam 1, and preliminary observations indicated that the structural response obtained during the initial loading stages influenced the ability of the BOTDA system to capture strain evolution with sufficient resolution. In addition, the two beams had different compressive strengths, resulting in variations in stiffness and cracking behavior. Therefore, the loading protocol for Beam 1 was adjusted by adopting a lower target load in the first cycle and progressively increasing load amplitudes in subsequent cycles with controlled incremental stages to facilitate observation of the beam response. This modification did not alter the overall four-point bending configuration or boundary conditions but was intended to enhance strain measurement accuracy and data interpretation. The loading-unloading cycles were continued progressively until flexural cracking developed and ultimate failure occurred in the mid-span region.

**Fig. 5:**
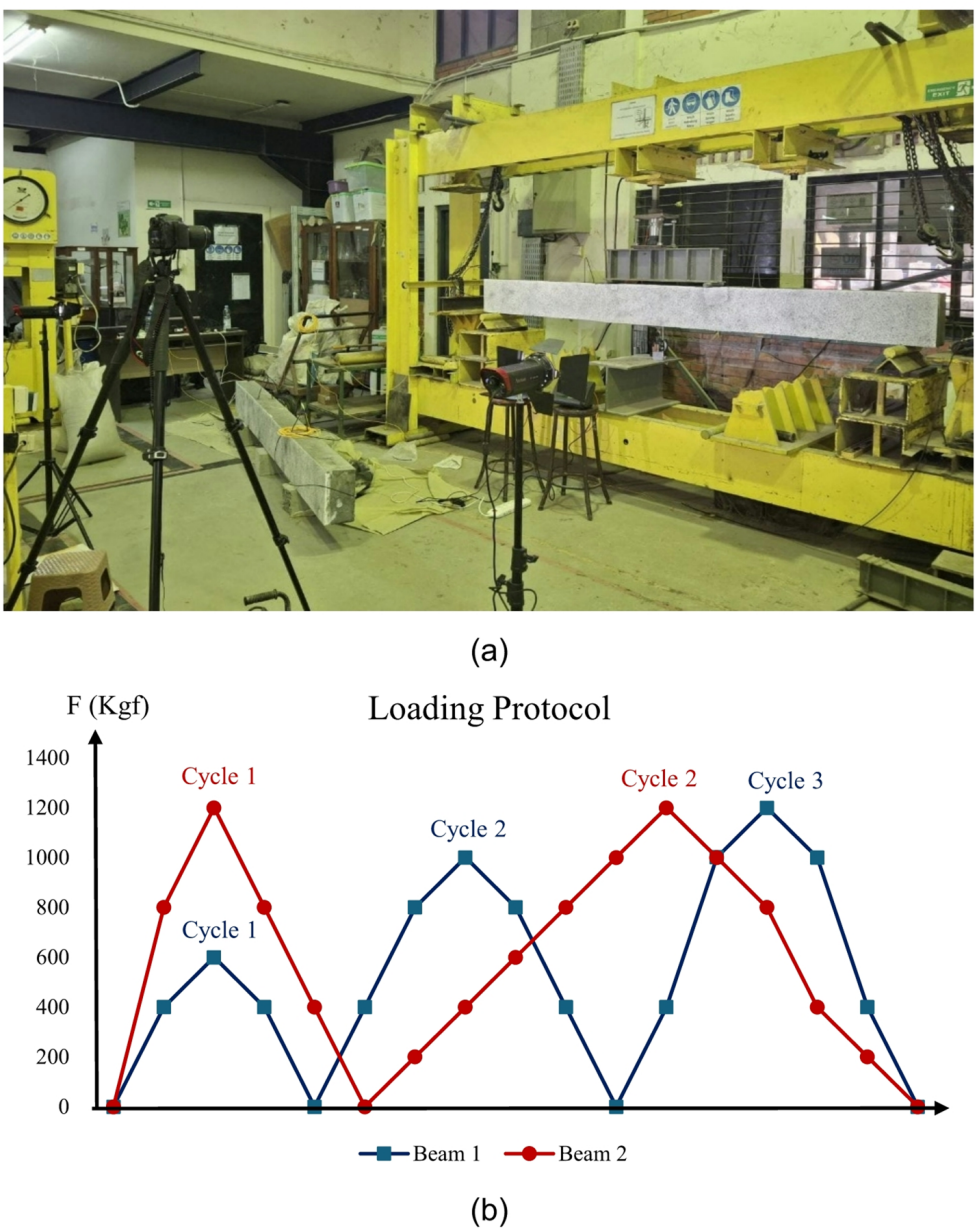
Experimental program: (**a**) four-point bending test setup with DIC camera and instrumentation, and (**b**) cyclic loading protocol applied to Beam 1 and Beam 2.

To record vertical displacements, a Linear Variable Differential Transformer (LVDT) was installed at the mid-span soffit of the beam to monitor deflection. The LVDT readings were used as reference data for validating the displacements reconstructed from BOTDA and DIC measurements. Additional dial gauges were positioned near the supports to monitor any vertical movement and ensure stable boundary conditions during the entire test. The overall test setup, including the loading arrangement, measurement instrumentation, and loading protocol, is illustrated in Fig. 5.

### BOTDA measurement and strain deflection reconstruction

The distributed strain measurements along the RC beams were obtained using the Brillouin Optical Time Domain Analysis (BOTDA) sensing system attached on the reinforcing bars. The BOTDA system operates on the principle of stimulated Brillouin scattering (SBS) in optical fibers, where the Brillouin frequency shift (ΔνB) varies linearly with the applied strain (ε) along the sensing fiber. The relationship can be expressed as:

$$\Delta_{\mathrm{VB}}{=\:}{\mathrm{C}}_{\varepsilon} \cdot \varepsilon$$

where $\:{\mathrm{C}}_{\varepsilon}$ is the strain sensitivity coefficient. In this study, a calibration value of $\:{\mathrm{C}}_{\varepsilon}$ = 0.05 MHz/µε was used to convert the frequency shift into distributed strain.

Two single-mode optical fibers were bonded longitudinally to the top and bottom mid longitudinal reinforcing bars of each beam to monitor strain in the compression and tension zones, respectively (see Fig. 1b). The optical fibers were installed along the longitudinal reinforcement bars and mechanically fixed using cable ties prior to concrete casting. This installation ensured that the optical fibers followed the strain development of the reinforcement during flexural loading. The BOTDA system provided a spatial resolution of 8 cm, resulting in discrete strain readings along the beam length. Due to this finite spatial resolution, the strain measured by the BOTDA system represents an average value over 8 cm gauge length rather than the localized peak strain at a specific location. Consequently, highly localized strain concentrations associated with flexural cracking may not be captured directly but are reflected through their influence on the surrounding strain field within the measurement length.

The distributed strain data from the top (ε-top) and bottom (ε-bottom) fibers were processed to determine the curvature distribution (κ) along the beam using the following relationship^20^:

$$\:\kappa \mathrm{(x)\:}{=}\frac{{\epsilon\:}_{top}\left(x\right)-{\epsilon\:}_{bot}\left(x\right)}{d}$$

where $\:d$ is the effective distance between the top and bottom fibers (approximately 200 mm). The deflection profile $\:w\left(x\right)$ was reconstructed by double numerical integration of the curvature as follows^20^:

$$\:\mathrm{w(x)\:}{=}{\int\:}_{0}^{x}{\int\:}_{0}^{\xi\:}{\kappa(}\eta\:)\:d\eta\:\:d\xi\:+{C}_{1}x+{C}_{2}$$

Boundary conditions $\:w\left(0\right)=w\left(L\right)=0$ were applied to satisfy the simply supported setup. Prior to numerical integration, the curvature data were smoothed using a Savitzky-Golay filter with a window size of 11 data points and a second-order polynomial to minimize numerical noise. The deflection profile was then reconstructed by double numerical integration of the smoothed curvature data, with boundary conditions corresponding to a simply supported beam configuration. The mid-span LVDT was used as an independent reference measurement to validate the deflection reconstructed from BOTDA strain data. The deflection derived from BOTDA was obtained directly from the strain-based formulation without any adjustment to match the LVDT measurements.

All BOTDA data acquisition and processing were performed using a single-channel optical sensing unit with a Brillouin gain spectrum (BGS). The resulting distributed strain and reconstructed deflection data were subsequently compared with measurements obtained from Digital Image Correlation (DIC) and LVDT systems.

### DIC measurement

In addition to BOTDA, Digital Image Correlation (DIC) was employed to obtain full-field displacement and strain data on the beam surface during the four-point bending test. The DIC technique is a non-contact optical method that tracks pixel intensity variations between reference and deformed images to measure displacement fields with sub-pixel accuracy.

A Canon EOS 80D digital camera was used for image acquisition. The camera was positioned perpendicular to the beam’s side surface at a distance of approximately 2.5 m to cover the entire span within the field of view. The Canon 80D, equipped with a 24.2-megapixel CMOS sensor and EF-S 18–135 mm lens, was operated at a frame rate of 3 frames per second (fps) with an image resolution of 5472 × 3648 pixels. The camera was mounted on a rigid tripod, and two LED light panels were arranged laterally to provide uniform illumination and minimize shadow effects during loading.

Prior to testing, the beam surface was prepared with a random black-and-white speckle pattern to enhance texture contrast for image correlation. The pattern was created using matte white paint as a base layer, followed by random black spray spots, resulting in a mean speckle size of approximately 3–5 pixels, which meets the optimal correlation criterion. A scale marker was attached to the beam to convert pixel displacement into physical units (mm).

The captured image sequences were processed using NCorr, an open-source DIC software implemented in MATLAB. A subset size of 35 pixels and a step size of 5 pixels were selected based on the pixel-to-mm calibration ratio to balance spatial resolution and computational efficiency. The software computed displacement fields in both the horizontal (u) and vertical (v) directions, from which strain maps were derived using finite-difference methods. The vertical displacement at the mid-span was extracted from the DIC data to validate the deflection reconstructed from BOTDA and LVDT measurements.

Figure 5(a) illustrates the DIC system configuration, the speckle pattern applied for surface preparation, and the loading protocol adopted for the experimental program.

## Results and discussion

### Load–displacement behavior

**Fig. 6:**
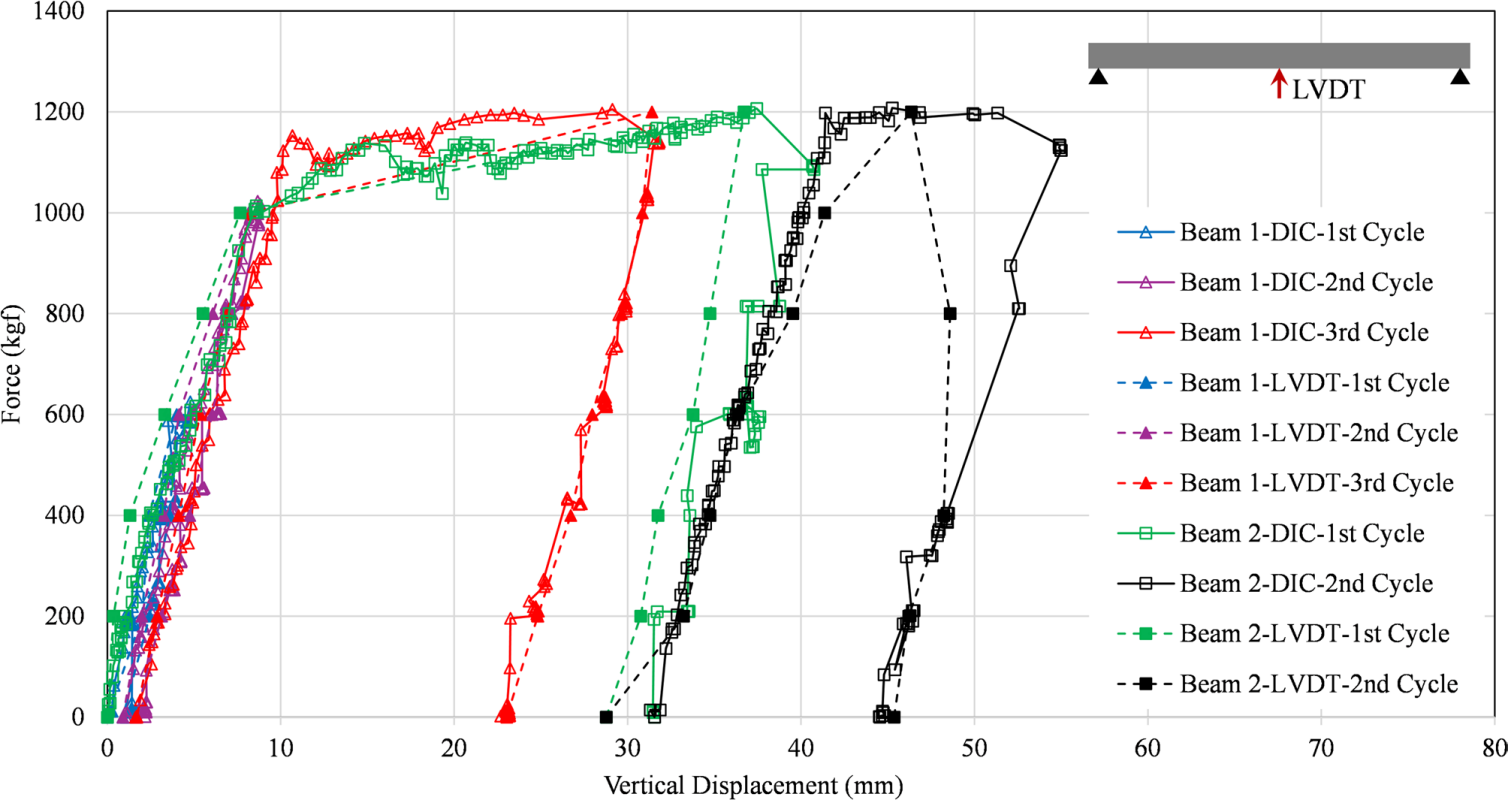
Load-displacement relationship of RC Beam 1 and 2 obtained from mid-span LVDT and DIC measurements. Beam 1 was tested under three loading cycles (Cycles 1–3), while Beam 2 was tested under two loading cycles (Cycles 1–2).

The global load-displacement behavior of the RC beams obtained from LVDT measurements at the mid-span is shown in Fig. 6 for Beam 1 and Beam 2, respectively. Both specimens exhibited a typical flexural response under four-point bending, characterized by an initial linear elastic phase followed by gradual stiffness reduction and increased deflection until failure. The maximum applied load for both beams was approximately 1200 kgf, corresponding to the flexural capacity of the CP19S1 concrete mix. It should be noted that Beam 1 and Beam 2 had different concrete compressive strengths due to variations in environmental conditions during casting and early curing; therefore, the two specimens represent distinct material conditions rather than directly comparable structural replicates.

As illustrated in Fig. 6, Beam 1 was subjected to three successive loading cycles, consisting of loading, partial unloading, and reloading to higher levels. During the first cycle, the beam behaved linearly up to around 400 kgf without visible cracking. The second cycle showed minor stiffness degradation due to the initiation of flexural cracks near mid-span. In the third cycle, the load increased to approximately 1200 kgf, resulting in a rapid rise in deflection up to 30 mm before failure occurred. Residual displacement after unloading indicated the presence of permanent deformation caused by crack propagation and microstructural damage in the tension zone.

Meanwhile, as presented in Fig. 6, Beam 2 experienced two loading cycles following the same four-point bending procedure. The first cycle was terminated after the initial crack formation, while the second was continued until failure. The maximum load also reached approximately 1200 kgf, but the beam exhibited a larger ultimate deflection of about 50 mm, reflecting differences in structural response associated with the variation in concrete compressive strength. The difference in displacement behavior between the two beams is mainly attributed to variations in concrete batch strength and the crack development rate.

Overall, Fig. 6 demonstrates a consistent flexural failure mechanism dominated by tensile cracking in the constant-moment region. The progressive reduction in stiffness across loading cycles highlights the nonlinear flexural response typical of reinforced concrete beams under repeated loading. These results, derived from LVDT measurements, serve as the reference deflection data for validating the displacement responses obtained from BOTDA and DIC in the following sections.

### DIC results and longitudinal deformation visualization

**Fig. 7:**
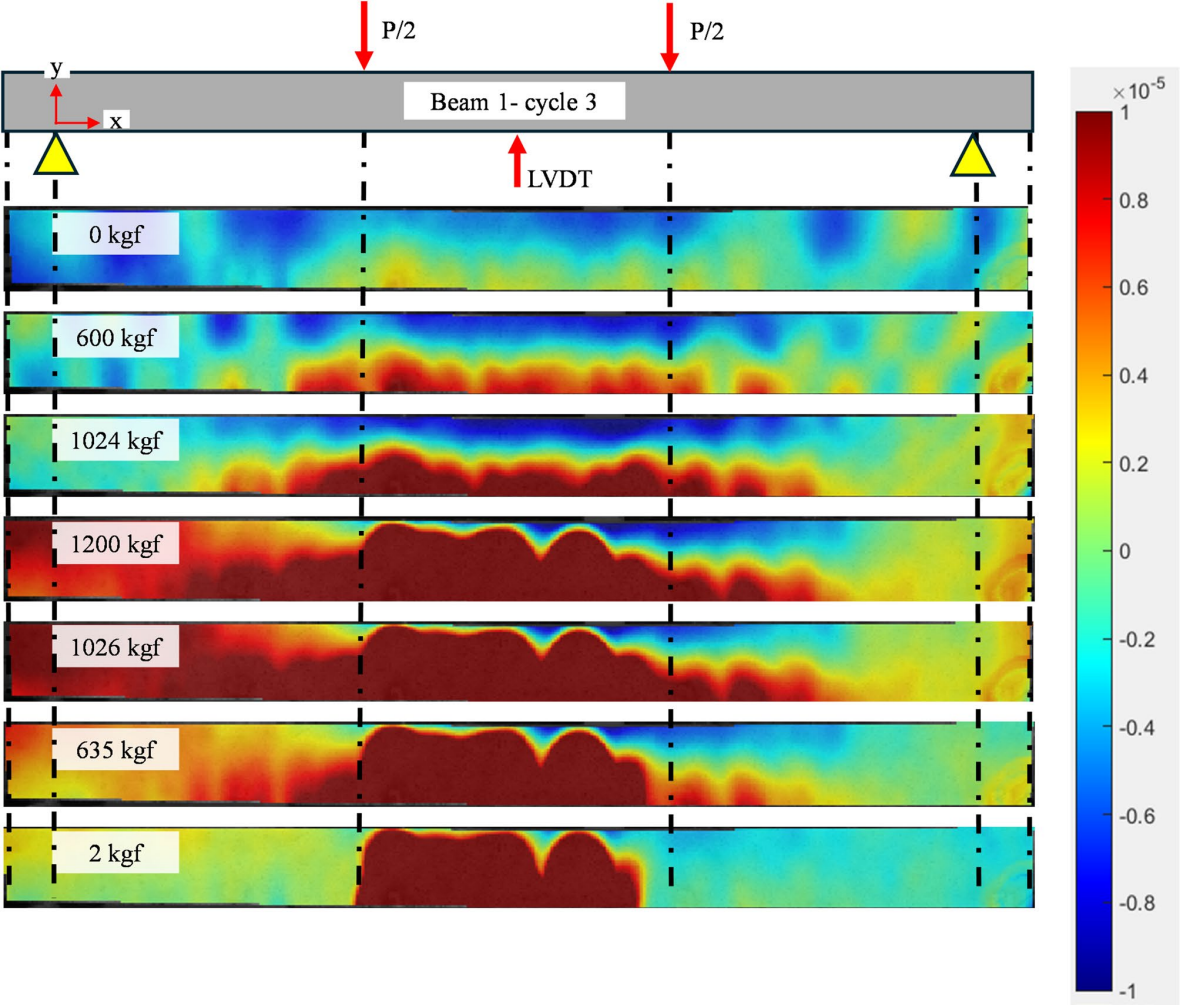
Longitudinal strain distribution (*ε*_*xx*_) of RC Beam 1 during the third loading cycle obtained from DIC analysis using NCorr.

**Fig. 8:**
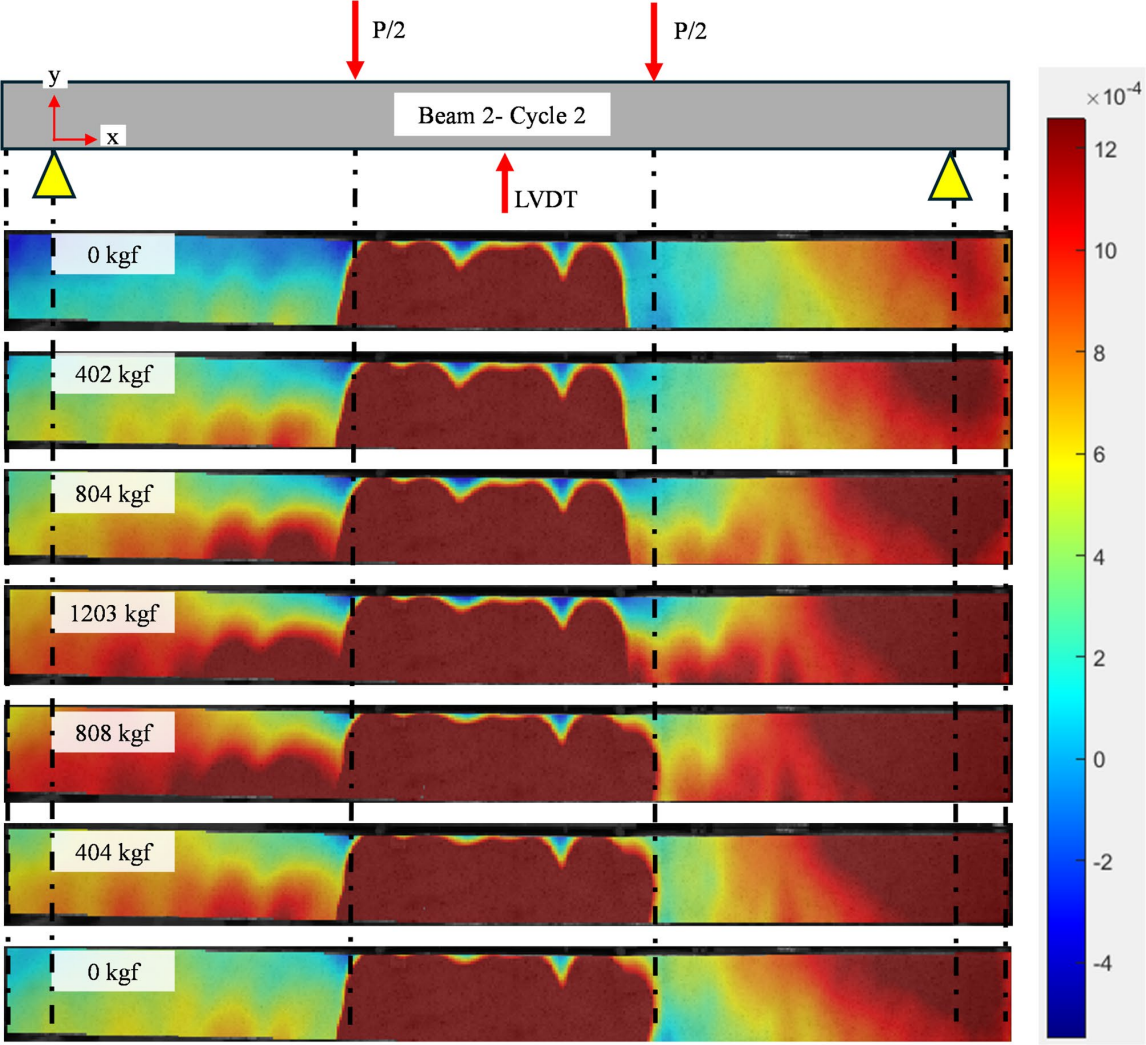
Longitudinal strain distribution (*ε*_*xx*_) of RC Beam 2 during the second loading cycle obtained from DIC analysis using NCorr.

The longitudinal strain distributions (*ε*_*xx*_) obtained from Digital Image Correlation (DIC) provide full-field visualization of the deformation behavior of both RC beams during the four-point bending test. Considering the different concrete compressive strengths of the two specimens, the observed deformation patterns reflect their distinct structural responses under flexural loading. The processed strain maps for Beam 1 and Beam 2, corresponding to their final loading cycles, are presented in Figs. 7 and 8, respectively. The results clearly demonstrate the evolution of flexural deformation along the beam span under increasing load levels.

As shown in Fig. 7, the strain field of Beam 1 during the third loading cycle developed progressively as the applied load increased from 0 to 1200 kgf. At the early stages (0–600 kgf), the strain was uniformly distributed, indicating an elastic response of the beam. As the load increased to 1024–1200 kgf, pronounced strain concentrations emerged in the mid-span region, representing the initiation and propagation of flexural cracks within the constant-moment zone. The red regions in the contour maps indicate high tensile strain near the bottom fiber, while the blue regions correspond to compressive strain near the top fiber. The subsequent unloading stages (1026–2 kgf) show partial recovery of the strain field, leaving visible residual deformation, which confirms permanent microcrack development in the tension zone.

Similarly, Fig. 8 presents the strain maps for Beam 2 during the second loading cycle. The beam exhibited a comparable deformation pattern, with strain localization beginning at approximately 400 kgf and intensifying as the load increased to 1203 kgf. The concentration of tensile strain was also centered in the mid-span region, demonstrating symmetrical flexural behavior consistent with the four-point bending configuration. Beam 2 exhibited a larger strain magnitude at the bottom fiber, which is consistent with its observed deflection behavior in the load–displacement response (Fig. 6) and can be attributed primarily to differences in concrete strength and resulting stiffness characteristics.

The DIC analysis confirms that both beams experienced dominant flexural deformation with negligible shear effects, as evidenced by the uniform strain bands across the constant-moment zone. The symmetry of the deformation patterns further validates the accuracy of the experimental setup and the boundary conditions. These results demonstrate that the DIC system effectively captured flexural strain evolution in both specimens despite their different material properties. These DIC strain results will later be compared with the distributed strain measurements obtained from the BOTDA system to evaluate their correlation in capturing the flexural behavior of the RC beams.

### Horizontal displacement and crack width estimation from DIC

**Fig. 9:**
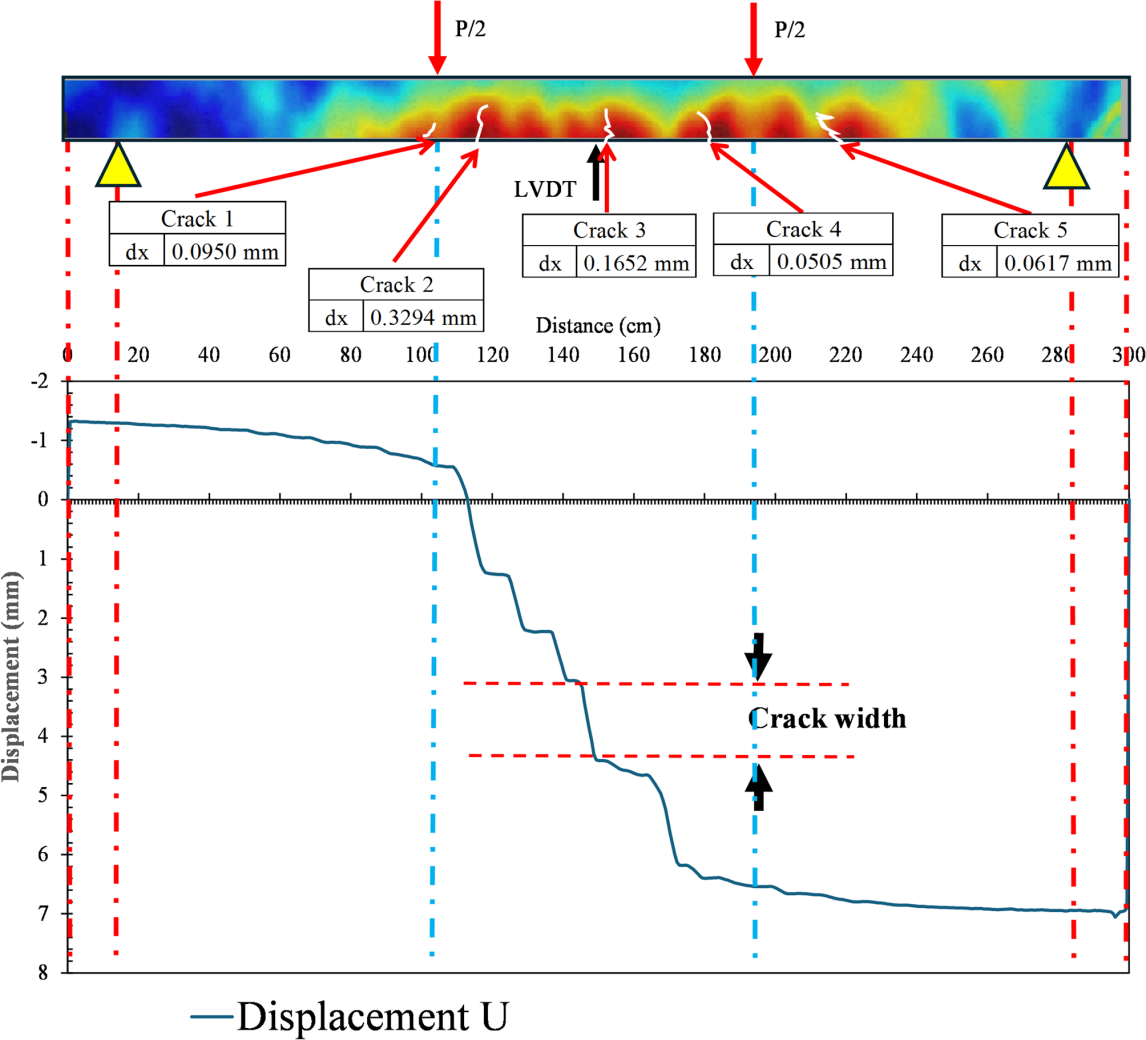
Horizontal displacement distribution (U) and crack-width estimation of RC Beam 1 at the final loading cycle obtained from DIC analysis.

**Fig. 10:**
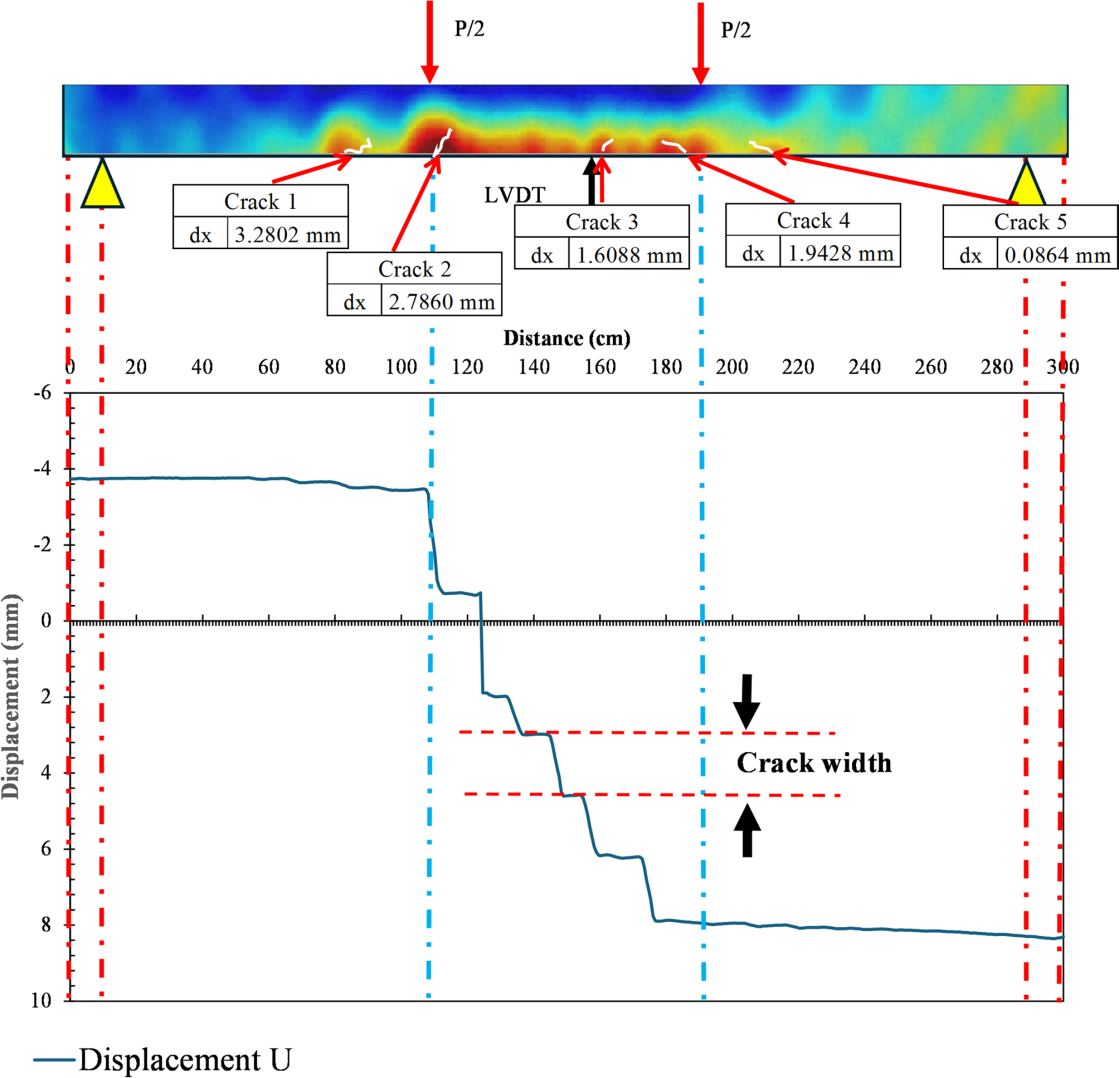
Horizontal displacement distribution (U) and crack-width estimation of RC Beam 2 at the final loading cycle obtained from DIC analysis.

The horizontal displacement fields obtained from the Digital Image Correlation (DIC) analysis were used to quantify the crack openings along the beam surface at the final loading cycle. The contour maps and displacement-distance plots for Beam 1 and Beam 2 are shown in Figs. 9 and 10, respectively. Considering the different concrete compressive strengths of the two specimens, the crack characteristics presented here reflect their distinct structural responses under flexural loading. The horizontal displacement (*U*) corresponds to the displacement along the longitudinal axis of the beam, extracted from the DIC displacement field to identify discontinuities associated with flexural crack openings.

As presented in Fig. 9, Beam 1 exhibited five distinct cracks distributed symmetrically along the constant-moment region. The estimated crack widths (*dx*) were 0.0950 mm, 0.3294 mm, 0.1652 mm, 0.0505 mm, and 0.0617 mm, measured from left to right along the span. The largest crack opening (0.3294 mm) occurred near the mid-span, which corresponds to the maximum bending moment zone and agrees with the high-strain region observed in the DIC longitudinal strain contours (Fig. 7). The displacement profile below the contour plot shows clear step-like discontinuities, representing localized deformation across each crack plane.

Similarly, Fig. 10 shows the horizontal displacement distribution for Beam 2 at the ultimate load stage. Five major cracks were identified with corresponding crack widths of 3.2802 mm, 2.7860 mm, 1.6088 mm, 1.9428 mm, and 0.0864 mm. Beam 2 exhibited significantly larger crack openings, which can be attributed primarily to differences in concrete compressive strength and the associated stiffness and cracking behavior. The crack localization remained concentrated around the mid-span region, consistent with the theoretical constant-moment area of the four-point bending test.

The DIC-based crack width estimation demonstrates the ability of this optical technique to detect and quantify local deformation with sub-millimeter accuracy. The observed crack spacing and magnitude provide essential validation for the distributed strain profiles obtained from the BOTDA measurements discussed in “Deflection reconstruction based on BOTDA strain data”. Overall, the displacement discontinuities observed in both beams confirm that flexural cracking governed the failure mechanism, with negligible shear deformation.

Due to its finite spatial resolution, the BOTDA system provides strain values averaged over the gauge length and cannot directly resolve highly localized strain concentrations associated with flexural cracks. The crack widths observed in reinforced concrete beams are typically smaller than the 8 cm spatial resolution, and therefore BOTDA measurements represent the influence of cracking on the surrounding strain field rather than the true peak strain at the crack plane or the crack opening displacement itself. Consequently, BOTDA is not suitable for direct crack width measurement. In this study, Digital Image Correlation (DIC) was employed to quantify surface crack openings, while BOTDA was used to capture the distributed strain evolution associated with the global flexural behavior of beams. Although a Savitzky–Golay filter was applied to reduce numerical noise prior to deflection reconstruction, crack opening measurements were obtained using DIC, while BOTDA was used to capture the distributed strain evolution associated with the global flexural behavior of the beam.

### BOTDA distributed strain analysis

The distributed strain profiles obtained from the Brillouin Optical Time Domain Analysis (BOTDA) system provide a clear representation of the flexural response of the RC beams under four-point bending. BOTDA measurements were taken along both the top (compression) and bottom (tension) fibers of the beams with a spatial resolution of approximately 8 cm, allowing continuous strain monitoring across the entire 3 m span. The results presented in this section correspond to the final loading cycles of each beam (Cycle 3 for Beam 1 and Cycle 2 for Beam 2), which represent the critical deformation stages before failure. The distributed strain responses discussed below reflect their distinct structural behaviors under flexural loading.

**Fig. 11:**
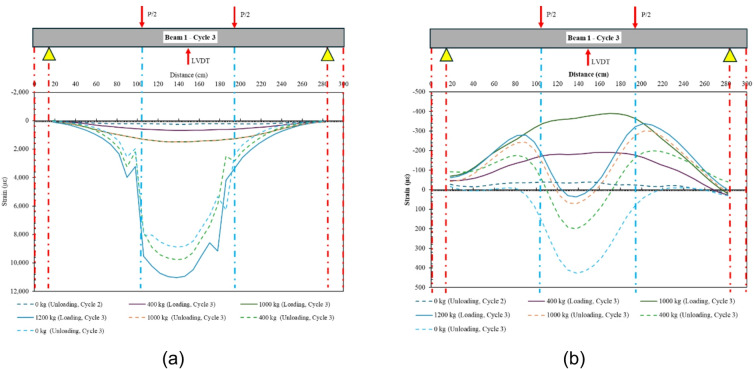
Distributed tensile strain profiles along the RC Beam 1 during the third loading cycle obtained from BOTDA measurements: (**a**) Bottom fiber and (**b**) Top fiber.

Figure 11a,b present the distributed strain profiles for the bottom and top fibers of Beam 1 at different load levels (0, 400, 1000, and 1200 kgf). The dashed lines indicate the effective optical fiber sensing length (280 cm), which is shorter than the total beam length (300 cm). At the early load stages (400 kgf), both fibers exhibited nearly linear and symmetric strain distributions, with maximum magnitudes below 200 µε, indicating elastic behavior and no cracking in the tension zone.

As the load increased to 1000 kgf and 1200 kgf, the bottom-fiber strain (*ε*_*bottom*_) increased rapidly, reaching values of approximately 8000–10 000 µε in the mid-span region. This sharp rise in tensile strain signifies the development and propagation of flexural cracks in the constant-moment zone. Meanwhile, the top-fiber strain (*ε*_*top*_) remained compressive with peak values of around − 400 µε, consistent with expected flexural behavior. The symmetrical pattern of strain about the mid-span indicates that the boundary conditions and loading setup were properly aligned, and that the beam response was dominated by bending rather than shear effects.

The strain value at 0 kg represents residual strain measured after gradual unloading and does not correspond to an absolute zero-strain reference. Therefore, non-zero strain values are observed at the unloaded stage.

The evolution of the strain profile from elastic to nonlinear clearly demonstrates the capability of BOTDA in capturing the evolution of distributed strain associated with flexural behavior. The mid-span strain concentration aligns with the high-strain regions identified from the DIC analysis, confirming the consistency between both optical sensing techniques.

**Fig. 12:**
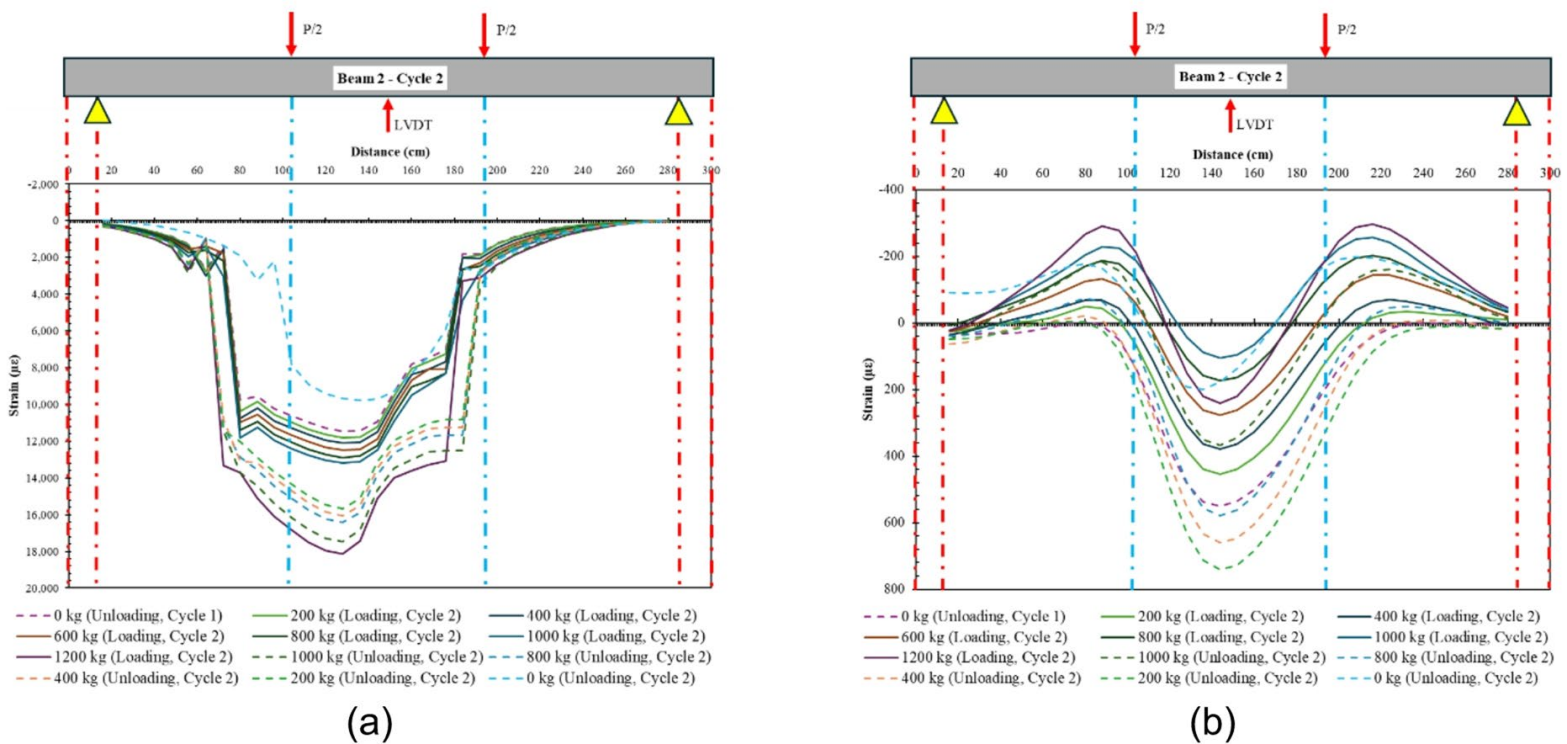
Distributed tensile strain profiles along the RC beam 2 during the second loading cycle obtained from BOTDA measurements: (**a**) Bottom fiber and (**b**) Top Fiber.

Figure 12 distributed strain profiles measured by BOTDA along the reinforcement of Beam 2: (a) tensile strain in the bottom reinforcement fiber, and (b) compressive strain in the top reinforcement fiber at different loading stages corresponding to load levels of 0, 200, 400, 600, 800, 1000, and 1200 kgf. A similar flexural trend was observed, with the *ε*_*bottom*_ values increasing significantly with load. At 1200 kgf, the bottom fiber exhibited tensile strain peaks of about 15 000–18 000 µε in the mid-span region reflecting differences in structural response associated primarily with the variation in concrete strength and cracking behavior.

The *ε*_*top*_ values remained compressive, ranging between − 200 µε and − 600 µε, while the bottom fiber strain reached an order of magnitude higher, further confirming that the failure mode was dominated by flexural cracking. The strain profiles were symmetric and concentrated in the constant-moment zone, consistent with the theoretical bending moment diagram and the DIC strain patterns shown in Figs. 11 and 12.

The larger tensile strain values recorded in Beam 2 indicate more pronounced cracking and greater inelastic deformation prior to failure, consistent with its observed deflection behavior in the load-displacement response (Fig. 6). This behavior is associated with the lower compressive strength of Beam 2 (25.41 MPa), which resulted in a less confined compression zone and earlier crack propagation, allowing larger tensile strain development in the reinforcement before failure.

These results demonstrate that the BOTDA system could capture distributed strain evolution in specimens with different material strengths, confirming its applicability under varying structural conditions.

### Deflection reconstruction based on BOTDA strain data

**Fig. 13:**
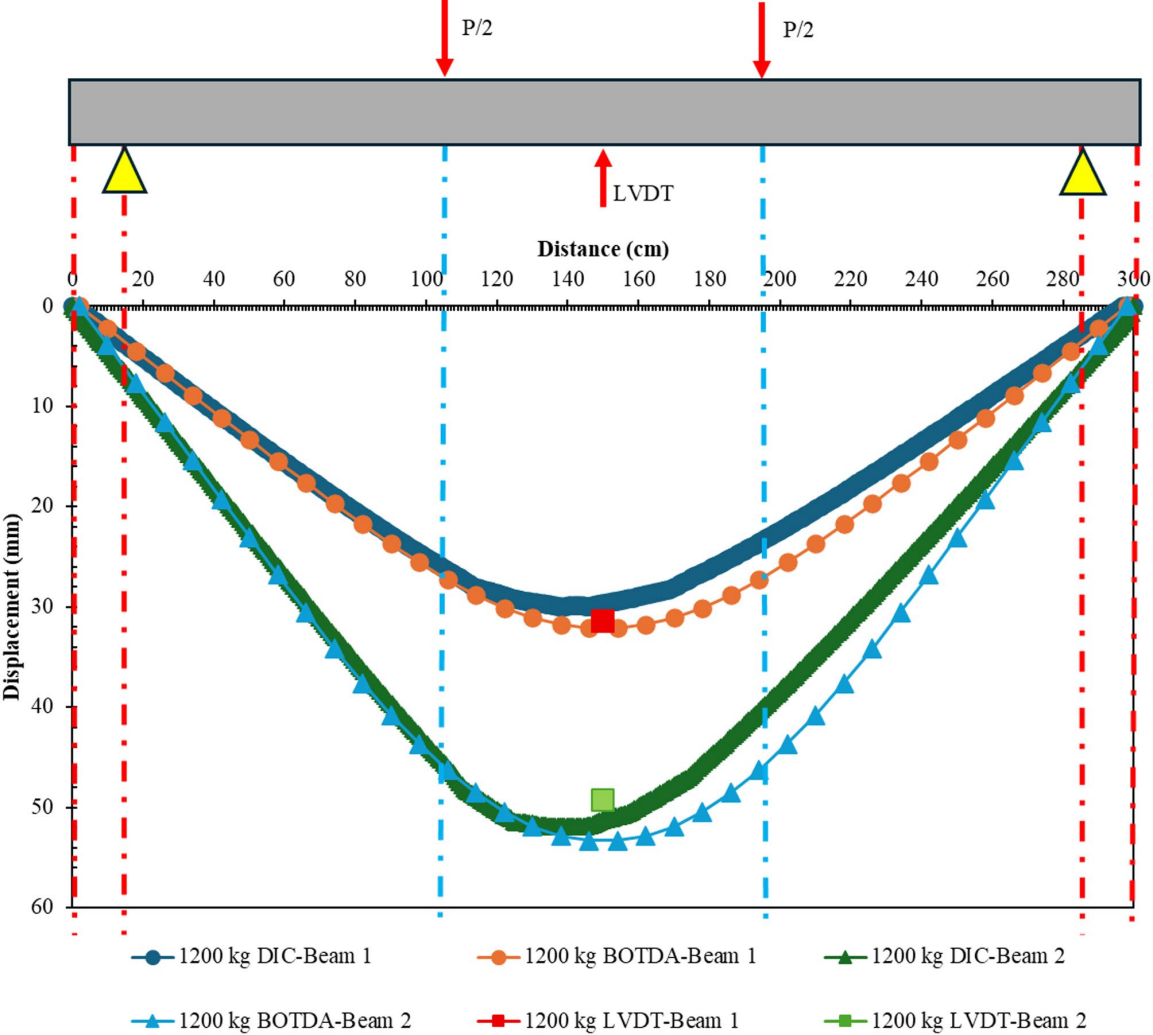
Comparison of deflection profiles obtained from BOTDA, DIC, and LVDT for RC Beam 1 and Beam 2 at the maximum load of 1200 kgf.

The deflection profiles of both RC beams were reconstructed from the distributed strain results obtained using the BOTDA system. The reconstructed deflections obtained from BOTDA strain data were compared with those measured by the DIC and LVDT systems at the peak load of 1200 kgf, as shown in Fig. 13. The reconstructed deflection responses reflect their distinct structural behaviors under flexural loading.

The reconstructed curves for Beam 1 and Beam 2 exhibit smooth and symmetrical shapes consistent with the theoretical flexural behavior of simply supported beams under four-point bending. The BOTDA-derived deflection profiles closely followed the LVDT and DIC results along the entire span. It should be noted that the BOTDA deflection was derived independently from the strain-based formulation, while the LVDT measurements were used solely for validation. The largest deflection differences were found near the mid-span, with discrepancies generally below 7%, indicating good agreement among the three measurement systems.

For Beam 1, the maximum deflection recorded by BOTDA reached approximately 28 mm, compared to 29 mm from DIC and 30 mm from LVDT. For Beam 2, the maximum deflection values were 47 mm, 48 mm, and 50 mm, respectively. The larger deflection in Beam 2 is consistent with the higher tensile strain magnitudes recorded by BOTDA and reflects differences in structural response associated with the variation in concrete compressive strength.

The BOTDA system measures strain as an average over its spatial resolution and therefore cannot capture the true peak strain or crack opening at localized crack planes. Consequently, BOTDA is not suitable for direct crack width measurement. In this study, crack widths were quantified using DIC, while BOTDA provided distributed strain information reflecting global flexural behavior.

**Table 2:** Comparison of maximum deflection and percentage difference between BOTDA, DIC, and LVDT for RC Beam 1 and Beam 2.

Beam	Method	w_max_ (mm)	Difference from LVDT (%)
Beam 1	LVDT	30	–
	DIC	29.1	3
	BOTDA	28	6.7
Beam 2	LVDT	50	–
	DIC	48.5	3
	BOTDA	47	6

Overall, the results confirm that BOTDA can accurately reconstruct the global deflection profile of reinforced concrete beams with different material strengths based on distributed strain data. While DIC provides high-resolution surface deformation and LVDT serves as a precise reference, BOTDA offers the advantage of distributed, full-span sensing, making it a powerful complement to conventional and optical field-based measurement methods.

These findings demonstrate that BOTDA remains effective for deflection monitoring across specimens exhibiting different structural responses, highlighting its applicability for practical structural health monitoring scenarios.

## Conclusion

This study demonstrated that both Brillouin Optical Time Domain Analysis (BOTDA) and Digital Image Correlation (DIC) are effective monitoring techniques for assessing the flexural response of RC beams subjected to four-point bending. The measured load-deflection behavior showed a nonlinear response with stiffness degradation and flexural cracking developing in the constant moment region. Considering that the two beams exhibited different concrete compressive strengths due to variations in casting and curing conditions, the observed structural responses represent distinct material behaviors rather than directly comparable replicates. BOTDA successfully captured the distributed strain profile along the beam and detected increasing tensile strain at the bottom fiber, reaching approximately 10,000 to 18,000 µε near ultimate load. The reconstructed deflection from BOTDA closely matched LVDT and DIC measurements, with discrepancies below 7%, indicating reliable deformation reconstruction. Meanwhile, DIC provided high-resolution surface deformation measurements and accurately quantified crack development, with widths ranging from 0.05 to 3.28 mm. Together, these findings show that BOTDA is well suited for global distributed sensing, while DIC excels in detailed crack and localized deformation visualization, making their integration highly complementary for structural monitoring.

The differences in deflection and tensile strain observed between the two beams were primarily associated with their different concrete compressive strengths, which influenced elastic stiffness, cracking behavior, and curvature development under flexural loading. The lower-strength specimen exhibited reduced stiffness and earlier cracking, resulting in larger deformation and strain demand prior to failure, whereas the higher-strength specimen reached its ultimate condition at a smaller deformation level. These observations further confirm that the sensing systems could capture structural responses under varying material conditions.

The results indicate that hybrid BOTDA-DIC monitoring has strong potential for use in laboratory testing, validation of numerical models, and long-term structural health monitoring of reinforced concrete systems. The ability to combine global strain tracking with localized crack detection provides richer information than conventional point-based instrumentation and supports more accurate assessment of serviceability and damage progression. The successful performance observed in both specimens also demonstrates the viability of the eco-concrete mix also demonstrates its viability for use in full-scale structural elements.

Several constraints should be acknowledged. BOTDA resolution limits may reduce sensitivity to closely spaced cracks, and strain-to-deflection reconstruction requires careful processing. DIC performance depends on adequate camera positioning, surface preparation, and lighting conditions. Additionally, the study considered only monotonic loading and a limited number of specimens, which restricts statistical generalization.

Future studies may incorporate additional optical fibers bonded to the bottom concrete cover to directly capture crack initiation and propagation. Further research should also investigate cyclic and fatigue loading conditions, long-term sensor durability, and alternative embedding or bonding strategies to improve BOTDA crack detection sensitivity. Expanding the experimental scope to different beam geometries, reinforcement ratios, and eco-concrete formulations, as well as integrating complementary sensing techniques such as FBG, acoustic emission, or 3D-DIC, would support broader validation of the proposed monitoring approach.

In summary, the combined use of BOTDA and DIC provides a powerful and complementary sensing framework capable of bridging global deformation measurements and detailed crack behavior, offering significant potential for future experimental mechanics and structural health monitoring applications in reinforced concrete structures.

## Data Availability

The datasets generated and analyzed during the current study are available from the corresponding author upon reasonable request. Raw BOTDA and DIC sensing data, processed strain datasets, and supporting experimental documentation are archived at Universitas Indonesia.
